# Wirksamkeit in Deutschland verfügbarer internetbasierter Interventionen für Depressionen – ein systematisches Review mit Metaanalyse

**DOI:** 10.1007/s00115-023-01587-0

**Published:** 2024-01-23

**Authors:** Raoul Haaf, Pia Vock, Nikolaj Wächtershäuser, Christoph U. Correll, Stephan Köhler, Jan Philipp Klein

**Affiliations:** 1 Klinik für Psychiatrie und Psychotherapie, Charité – Universitätsmedizin Berlin, corporate member of Freie Universität Berlin and Humboldt-Universität zu Berlin, Campus Charité Mitte, Berlin, Deutschland; 2 Klinik für Psychiatrie, Psychosomatik und Psychotherapie, Universität zu Lübeck, Lübeck, Deutschland; 3 Klinik für Kinder- und Jugendpsychiatrie, Charité – Universitätsmedizin Berlin, Berlin, Deutschland; 4 Department of Psychiatry, The Zucker Hillside Hospital, Northwell Health, Glen Oaks, NY USA; 5 Department of Psychiatry and Molecular Medicine, Donald and Barbara Zucker School of Medicine at Hofstra/Northwell, Hempstead, NY USA; 6 Center for Brain, Behavior and Metabolism, Universität zu Lübeck, Lübeck, Deutschland

**Keywords:** Unipolare Depression, Digitale Gesundheitsanwendungen, Internet- und mobilbasierte Interventionen (IMI), Wirkstärke, Langzeiteffekte, Unipolar depression, Digital health applications, Internet and mobile-based interventions (IMI), Efficacy, Long-term effects

## Abstract

**Hintergrund:**

Internetbasierte Interventionen (IBIs) zur Behandlung von Depressionen zeigen in internationalen Metaanalysen positive Effekte. Es ist jedoch unklar, ob diese Effekte sich auch auf die in Deutschland verfügbaren IBIs erstrecken. Ziel dieser Metaanalyse war es, die unmittelbaren Effekte und die Langzeiteffekte der in Deutschland frei oder als sog. digitale Gesundheitsanwendungen (DiGA) auf Rezept verfügbaren IBIs abzuschätzen und die Wirkstärke von DiGA und frei verfügbaren IBIs zu vergleichen.

**Methode:**

Es erfolgte eine systematische Literaturrecherche und Random-effects-Metaanalyse (Präregistrierung: INPLASY202250070). Berücksichtigt wurden RCTs von in Deutschland frei verfügbaren oder als DiGA erhältlichen IBIs an Erwachsenen mit erhöhter depressiver Symptomatik im Vergleich zu aktiven und inaktiven Kontrollen zum Erhebungszeitpunkt im Mai 2022.

**Ergebnisse:**

Es wurden sechs Interventionen identifiziert: COGITO, deprexis, iFightDepression, moodgym, Novego und Selfapy. Die gepoolte Effektstärke von insgesamt 28 Studien mit 13.413 Teilnehmer*innen entsprach einem Effekt von Cohen’s *d* = 0,42, (95 %-Konfidenzintervall[KI]: [0,31; 0,54], *I*^*2*^ = 81 %). Die Analyse der Langzeiteffekte zeigte eine geringere Effektstärke von *d* = 0,29, (95 %-KI: [0,21; 0,37], *I*^*2*^ = 22 %, *n* = 10). Subgruppenanalysen deuteten auf eine mögliche Überlegenheit der drei im DiGA-Verzeichnis gelisteten Interventionen (*d* = 0,56, 95 %-KI: [0,38; 0,74], *I*^*2*^ = 83 %, *n* = 15) im Vergleich zu den drei frei verfügbaren Interventionen (*d* = 0,24, 95 %-KI: [0,14; 0,33], *I*^*2*^ = 44 %, *n* = 13) hin (*p* = *0,002*).

**Schlussfolgerung:**

Die in Deutschland verfügbaren IBIs für depressive Störungen sind wirksam und können daher in der therapeutischen Versorgung von Menschen mit depressiven Störungen eingesetzt werden. Möglicherweise sind nicht alle Interventionen gleich gut wirksam.

**Zusatzmaterial online:**

Die Online-Version dieses Beitrags (10.1007/s00115-023-01587-0) enthält ein umfangreiches Appendix mit weiterführenden Informationen zu den speziell hier untersuchten Interventionen (inkl. Links zu Herstellerwebsites/Verordnungsprocedere) sowie zu weiterführender Literatur. Anzahl und Zielrichtung der insgesamt verfügbaren Anwendungen, Listungsprozess und Verordnungsprozess der DiGA werden dort beschrieben.

## Abstract (en)

**Background:**

Internet-based interventions (IBIs) for the treatment of depression have been found to have positive effects in international meta-analyses; however, it is unclear whether these effects also extend to IBIs specifically available in Germany. The aim of this meta-analysis was to estimate the immediate effects and the long-term effects of IBIs available in Germany free of charge or available on prescription and covered by the public health insurances as so-called digital health applications (DiGAs) and to compare the efficacy of DiGAs and freely available IBIs.

**Method:**

A systematic literature search and random-effects meta-analysis were performed (preregistration: INPLASY202250070). Randomized controlled trials (RCTs) of IBIs freely available in Germany or as DiGA in adults with elevated depressive symptoms were compared with active and inactive controls available at the time of the survey in May 2022.

**Results:**

A total of six interventions were identified: COGITO, deprexis, iFightDepression, moodgym, Novego, and Selfapy. The pooled effect size of a total of 28 studies with 13,413 participants corresponded to an effect of Cohen’s *d* = 0.42, (95% confidence interval, CI: 0.31–0.54, *I*^*2*^ = 81%). The analysis of long-term effects showed a smaller effect size of *d* = 0.29, (95% CI: 0.21–0.37, *I*^*2*^ = 22%, *N* = 10). Subgroup analyses indicated a possible superiority of the three interventions listed in the DiGA directory (*d* = 0.56, 95% CI: 0.38–0.74, *I*^*2*^ = 83%, *N* = 15) compared to the three freely available IBIs (*d* = 0.24, 95% CI: 0.14–0.33, *I*^*2*^ = 44%, *N* = 13, *p* *=* 0.002).

**Conclusion:**

The IBIs for depressive disorders available in Germany are effective and can therefore be used in the treatment of people with a depressive disorder; however, it is possible that not all interventions are equally effective.

## Hintergrund

Depressive Erkrankungen sind mit einer 12-Monats-Prävalenz von 7,7 % [1] in Deutschland weit verbreitet und gehen mit einer erheblichen Reduktion der Lebensqualität und Leistungsfähigkeit sowie erheblichen volkswirtschaftlichen Auswirkungen einher [2]. Durch ihren niedrigschwelligen Zugang haben internetbasierte Interventionen (IBIs) das Potenzial, die Versorgungssituation von Patient*innen mit Depressionen zu verbessern [3]. Im Internet stehen > 10.000 Apps zum Themenbereich psychische Gesundheit zur Verfügung, die nur in seltenen Fällen evidenzbasiert sind [4, 5].

Davon abzugrenzen sind nach wissenschaftlichen Standards konzipierte und untersuchte IBIs zur Depressionsbehandlung, welche in Deutschland frei und kostenlos zur Verfügung stehen, oder als sog. digitale Gesundheitsanwendung (DiGA) bei entsprechender Diagnose und Indikation zulasten der gesetzlichen Krankenversicherung verordnet werden können [6]. Die Verordnung kann sowohl durch Ärzt*innen als auch durch psychologische Psychotherapeut*innen erfolgen; Patient*innen können sich beim Vorliegen einer entsprechenden Diagnose auch direkt an ihre Krankenkasse wenden und erhalten dann auch ohne Verordnung den Zugang zu einer DiGA.

Für die dauerhafte Aufnahme in das DiGA-Verzeichnis des Bundesamtes für Arzneimittel und Medizinprodukte (BfArM) muss der Nachweis sog. positiver Versorgungseffekte erbracht werden. Zu den positiven Versorgungseffekten zählen neben der Wirksamkeit auch sonstige Versorgungseffekte wie Erhöhung der Patientenzufriedenheit oder geringere Kosten durch Arbeitsunfähigkeit. Für eine vorläufige Aufnahme ins DiGA-Verzeichnis ist die Vorlage eines Evaluationskonzeptes ausreichend. Das bedeutet, dass nicht für alle im DiGA-Verzeichnis gelisteten IBIs in randomisierten Studien auf Wirksamkeit untersucht wurden. Insgesamt sind mehr als 50 IBIs im DiGA-Verzeichnis gelistet, etwa die Hälfte davon im Bereich der Behandlung psychischer Störungen [6].

Für detailliertere Informationen zu den Therapieprinzipien, Einsatzmöglichkeiten und den rechtlichen Rahmen der DiGA sei hier auf den eErgebnisteil 1.3 (Zusatzmaterial online) und weiterführende Literatur (z. B. [7]). verwiesen.

Internationale Metaanalysen zur Wirksamkeit von IBIs zur Behandlung von Depressionen im Vergleich zu Kontrollbedingungen zeigen insgesamt positive Effekte mit kleinen [8] bis mittleren [9] Effektstärken.

Obgleich IBIs bereits Eingang in die Empfehlungen der aktuellen S3-Leitlinie zur Behandlung der unipolaren Depression gefunden haben [10], wurde die Wirksamkeit der speziell in Deutschland verfügbaren Interventionen im Rahmen einer Metaanalyse noch nicht untersucht. In der vorliegenden Arbeit sollen daher die in Deutschland kostenfrei verfügbaren oder im DiGA-Verzeichnis gelisteten IBIs für depressive Störungen identifiziert werden, die in randomisiert-kontrollierten Studien (RCTs) getestet wurden, und hinsichtlich ihrer lang- und kurzfristigen Wirksamkeit im Vergleich zu Kontrollbedingungen untersucht werden; dabei untersuchen wir auch mögliche Wirksamkeitsunterschiede zwischen DiGA und frei verfügbaren IBIs.

## Methoden

### Studienselektion

Die Arbeit wurde gemäß den PRISMA-Kriterien durchgeführt und prospektiv registriert (INPLASY202250070). Die Identifizierung geeigneter Studien (eMethodenteil 1 Zusatzmaterial online) erfolgte mittels vorab definierter Suchstrategie auf PubMed, im DiGA-Verzeichnis, auf Webseiten von Interventionsherstellern sowie durch Expertenbefragung und war am 30.05.2022 abgeschlossen. Die Titel‑, Abstract- und Volltextsichtung potenzieller Studien erfolgte unter Zuhilfenahme der Review-Software Rayyan (HBKU Research Complex, Doha, Qatar; https://www.rayyan.ai) durch zwei unabhängige Reviewer (NW und PV). Eingeschlossen wurden RCTs, welche folgende Eigenschaften erfüllen:

- Veröffentlichung in Journalen mit einem Peer-Review-Verfahren,
- Teilnehmer*innen ab 18 Jahren mit durch validierte Fragebögen festgestellten erhöhten Depressionssymptomen,
- Randomisierung auf die Behandlung mit einer IBI und einer Kontrollbedingung.

Die untersuchte IBI musste auf Deutsch und in Deutschland als DiGA gelistet oder für die Allgemeinheit kostenfrei verfügbar sein.

Um eine umfassende Datenbasis zu gewährleisten, wurden auch Studien einbezogen, die die betreffenden IBIs in nichtdeutschen Populationen untersucht haben. Berücksichtigt wurden nur IBIs, deren Behandlungsschwerpunkt allein auf der Depression lag (nicht z. B. depressive Symptome speziell im Rahmen eines Diabetes), wobei Komorbiditäten zulässig waren. Um der realen Versorgungssituation in Deutschland Rechnung zu tragen, wurden Studien ausgeschlossen, welche eine Psychotherapie im persönlichen Kontakt als Kontrollbedingung nutzten und bei Studiendesigns mit mehreren Interventionsgruppen, die Daten jener Interventionsgruppe berücksichtigt, welche am ehesten (nach Ansicht der Reviewer) die aktuelle Versorgungssituation abbildet (z. B. wurden unbegleitete gegenüber therapeutisch begleiteten Interventionsgruppen in der Analyse bevorzugt).

Die untersuchte Zielgröße war die anhand einer validierten Skala erhobene depressive Symptomatik unmittelbar nach Ende der geplanten Nutzungszeit der Intervention sowie nach einem Follow-up-Zeitraum, sofern dieser berichtet wurde.

### Datenextraktion

Die Datenextraktion erfolgte durch zwei unabhängige Reviewer (NW und PV). Studiencharakteristika wurden dabei in vorab definierten Extraktionstabellen dokumentiert inklusive Metadaten (Autoren, Hersteller, Publikationsjahr), Methodik (Zielgröße, Zielpopulation, Einschlusskriterien, Kontrollgruppe, Vorhandensein und Art einer therapeutischen Begleitung, Durchführung einer Follow-up-Erhebung), Population (Stichprobenumfang, Alter, Anteil an weiblichen Personen, Ausgangsschwere der depressiven Symptomatik), Intervention (Name, psychotherapeutische Technik, Besonderheiten), Ergebnisse (Effektstärken nach Nutzungszeitraum der Intervention und ggf. nach Follow-up; eTabelle 3 Zusatzmaterial online). Wurden innerhalb einer Studie mehrere Primärmaße für die depressive Symptomatik berichtet, wurde für die Analyse jenes Maß gewählt, welches sowohl zum Postinterventionszeitpunkt als auch zum Follow-up-Zeitpunkt oder alternativ als erstes berichtet wurde. Lagen mehrere Follow-up-Erhebungszeitpunkte vor, wurde jeweils der erste Follow-up-Erhebungszeitpunkt für die statistische Analyse gewählt. Intention-to-treat(ITT)-Analysen wurden gegenüber Per-Protocol-Analysen bevorzugt.

Bei Studien, welche die Effektstärke Cohen’s *d* nicht berichteten, wurde Cohen’s *d *konsekutiv (1) aus der berichteten Effektstärke (wie bspw. Odds Ratio oder partielles η^2^) errechnet; (2) aus anderen Testgrößen wie bspw. einem F‑Wert oder t‑Wert errechnet; (3) basierend auf den verfügbaren Daten geschätzt; (4) falls Daten nicht erhältlich, Effektstärken ggf. aus anderen Metaanalysen übernommen. Effektgrößen von *d* = 0,2 galten dabei als gering, ab 0,5 als moderat und über 0,8 als groß [11]. Die Bewertung des Risikos einer Verzerrung wurde durch zwei unabhängige Reviewer (NW und PV) anhand der Kriterien des Cochrane Collaboration risk of bias assessment (RoB) tool [12] durchgeführt und anschließend miteinander verglichen. Unstimmigkeiten wurden durch Konsens geklärt.

### Statistische Analyse

Die statistischen Analysen wurden mittels der Software Review Manager (Version 5.4.1, Cochrane Collaboration) sowie mit SPSS Statistics (Version 28.0.1.1 [14], IBM) durchgeführt. Die Berechnung der gepoolten Effektschätzer mit 95 %-Konfidenzintervallen erfolgte mittels eines Random-effects-Modells. Für die grafische Darstellung der Ergebnisse wurde der Forest-Plot verwendet. Ein Funnel-Plot wurde zur Evaluation des Vorliegens eines Publikationsbias erstellt. Die Heterogenität wurde mit dem *I*^*2*^-Maß, die Symmetrie der Daten mit dem Egger-Test untersucht.

In einer Subgruppenanalyse für den Faktor DiGA (ja/nein) wurden die DiGA mit den frei verfügbaren IBIs sowohl zum Postinterventionszeitpunkt als auch zum Follow-up-Zeitpunkt miteinander verglichen. Zudem wurden mehrere Sensitivitätsanalysen durchgeführt, in deren Rahmen Studien mit folgenden Charakteristika ausgeschlossen wurden: 1) hohes Bias Risiko, 2) aktive Kontrollgruppe, 3) Population mit einer zusätzlichen komorbiden psychischen oder neurologischen Störung, und 4) nichtdeutschsprachige Zielpopulation.

## Ergebnisse

### Ergebnisse der Literatursuche

Die Ergebnisse der Literatursuche sind im PRISMA-Flussdiagramm online einsehbar (eAbbildung 1). Die Literatursuche ergab nach Eliminierung von Duplikaten 516 Treffer. Im Ergebnis der Titel-Abstract-Sichtung wurden 48 Publikationen in die Volltextsichtung einbezogen. 28 Publikationen erfüllten die Einschlusskriterien und wurden in die systematische Analyse eingeschlossen. In diesen Publikationen wurden sechs Interventionen untersucht:

- COGITO [13, 14],
- deprexis [15–25],
- iFightDepression [26],
- moodgym [27–36],
- Novego [37–39] und
- Selfapy [40].

Eine narrative und tabellarische Übersicht über die identifizierten Interventionen ist online einsehbar (eErgebnisteil 1.3). In ihren Grundzügen wiesen alle hier untersuchten Interventionen einen modularen Aufbau auf und enthielten zentrale Elemente der kognitiven Verhaltenstherapie wie Psychoedukation, kognitive Umstrukturierung und Verhaltensaktivierung. Die Module bestanden dabei meist aus einer Mischung von Texten, Animationen, Audios oder Videos und Übungen. Besonderheiten der einzelnen Interventionen zeigten sich beispielsweise in der Präsentation der Inhalte (simulierte Dialoge vs. einfache Fließtexte), der Dauer der Module (wenige Minuten bis eine Stunde) oder der empfohlenen Bearbeitungsreihenfolge (beliebige Reihenfolge vs. sequenziell).

### Studiencharakteristika

eTabelle 3 fasst die wichtigsten Charakteristika der eingeschlossenen Studien zusammen. Die Stichprobengröße der einzelnen Studien reichte von 26 bis 3805 Proband*innen und umfasste über alle Studien hinweg insgesamt 13.413 Personen mit einem mittleren Alter von 38,22 Jahren (SD = 10,61), von denen 74 % weiblich waren. Die depressive Symptomatik lag vor Interventionsbeginn im Mittel bei 89 % der Studien im moderaten bis schweren Bereich (übrige mild oder nicht angegeben). Alle Studien nutzten ausschließlich Selbstbeurteilungsfragebögen zur Erfassung der Depressivität (meist Beck-Depressionsinventar [BDI] oder Patient Health Questionnaire [PHQ]). Als Kontrollbedingungen wurden größtenteils Wartelistenbedingungen (64 %) eingesetzt, gefolgt von Regelbehandlung (20 %) und Übungsangeboten mit psychoedukativen bzw. achtsamkeitsfokussierten Elementen oder progressiver Muskelrelaxation (16 %). In 25 % der Studien wurde zusätzlich zur Intervention eine therapeutische Begleitung in Form kurzer Telefonate oder personalisierter E‑Mails angeboten.

Die durchschnittliche Interventionsdauer betrug 8,32 Wochen (SD = 3,29) und reichte von 3 bis 16 Wochen. Zum Postinterventionszeitpunkt lag die mittlere Drop-out-Rate der Interventionsgruppen bei 29 %, die der Kontrollgruppen bei 20 %. Von den 16 identifizierten Studien, die zusätzlich zum Postinterventionszeitpunkt eine Follow-up-Erhebung der depressiven Symptomatik durchführten, konnten 6 Studien aufgrund fehlender oder unpassender Daten nicht in die Analyse eingeschlossen werden (eErgebnisteil 1.2.4). Der durchschnittliche Follow-up-Erhebungszeitraum über die 10 verbleibenden Studien hinweg betrug 14,75 Wochen (SD = 6,54) nach Ende der Intervention.

### Risk-of-bias-Bewertung

**Figure 1:**
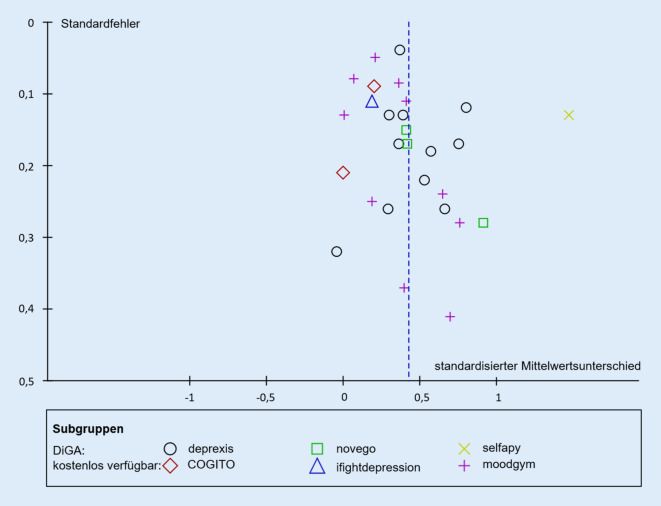

Keine der eingeschlossenen Studien wies ein niedriges Biasgesamtrisiko auf. Insgesamt wurden 17 Studien (61 %) mit einem mittleren und 11 Studien (39 %) mit einem hohen Biasrisiko bewertet (eErgebnisteil 1.2.3). Die visuelle Analyse des Funnel-Plots (Abb. 1) sowie der Egger-Test (*p* *=* *0,32*) ergaben keinen Hinweis auf das Vorliegen eines Publikationsbias.

### Effektivität der Interventionen

#### Postinterventionszeitpunkt

**Figure 2:**
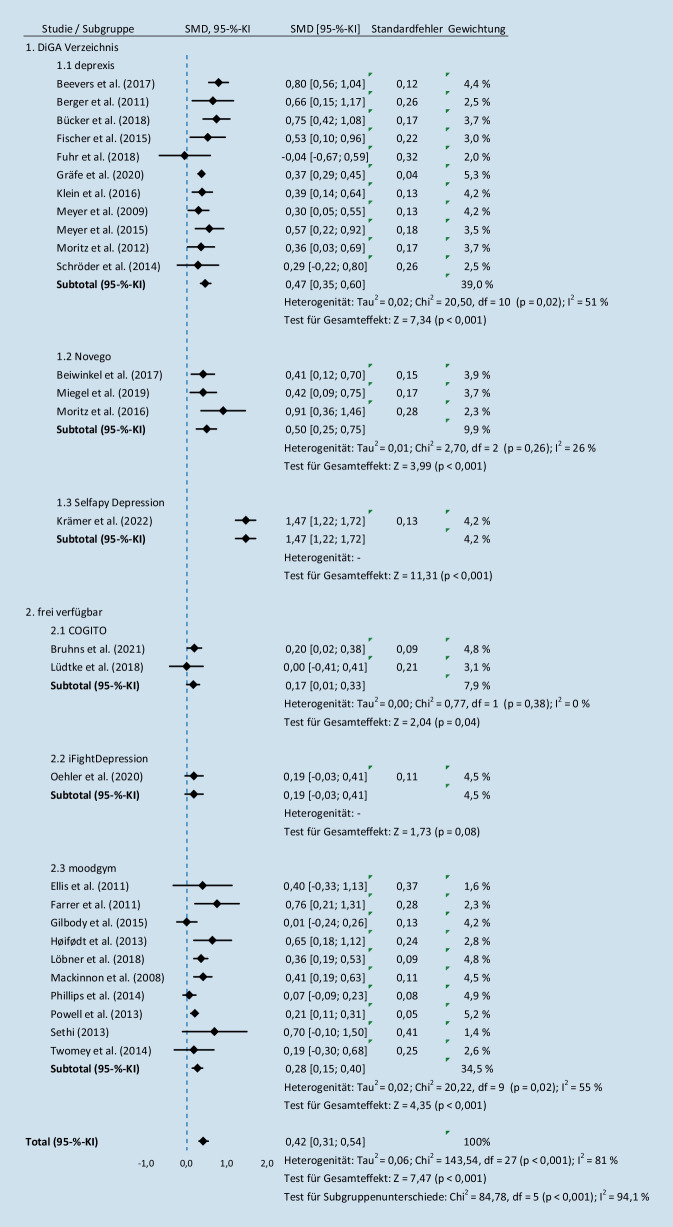

Zum Zeitpunkt nach Beendigung der geplanten Nutzungszeit (Postinterventionszeitpunkt) war die Behandlung mit einer IBI den Kontrollgruppen mit einem kleinen bis mittleren Effekt (*d* = 0,42, 95 %-KI: [0,31; 0,54], *I*^*2*^ = 81 %) überlegen (Abb. 2).

#### Follow-up-Erhebung

**Figure 3:**
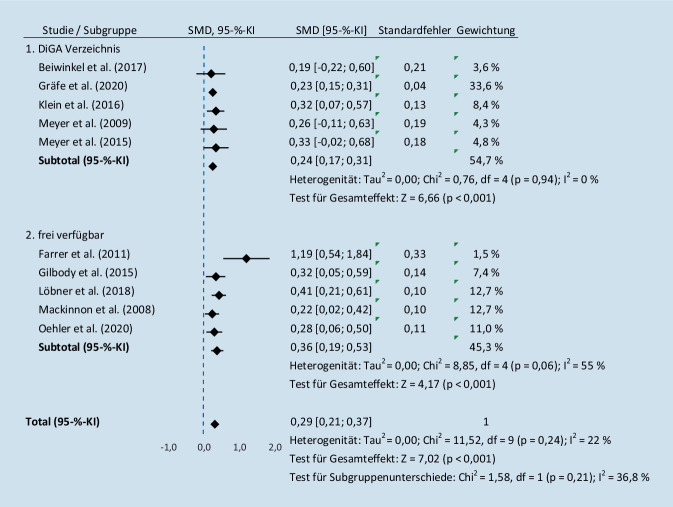

In der Follow-up-Analyse war die Behandlung mit einer IBI im Vergleich zu den Kotrollgruppen mit einem kleinen Effekt (*d* = 0,29, 95 %-KI: [0,21; 0,37], *I*^*2*^ = 22 %, *n* = 10) überlegen (Abb. 3).

### Subgruppenanalyse

Für den Faktor DiGA (ja/nein) zum Postinterventionszeitpunkt zeigte sich ein signifikanter Unterschied (*χ*^*2*^ = 9,58, df = 1, *p* *=* *0,002, I*^*2*^ = 89,6 %) zwischen der Gruppe der frei verfügbaren Interventionen (*d* = 0,24, 95 %-KI: [0,14; 0,33], *I*^*2*^ = 44 %) und der DiGA (*d* = 0,56, 95 %-KI: [0,38; 0,74], *I*^*2*^ = 83 %). Kein signifikanter Unterschied (*χ*^*2*^ = 1,38, df = 1, *p* *=* *0,24, I*^*2*^ = 27,5 %, *n* = 10) zeigte sich diesbezüglich bei der Follow-up-Analyse.

### Sensitivitätsanalysen

Die Ergebnisse der durchgeführten Sensitivitätsanalysen zum Postinterventionszeitpunkt waren alle vergleichbar mit den Ergebnissen der Hauptanalyse (eErgebnisteil 1.2.5).

## Diskussion

Die Ergebnisse der vorliegenden Metaanalyse weisen auf eine Überlegenheit der Behandlung mit den untersuchten IBIs gegenüber Kontrollbedingungen in der Reduktion depressiver Symptome hin, mit einer kleinen bis mittleren Effektstärke (*d* = 0,42) und anhaltenden Vorteilen bei Follow-up-Untersuchungen (*d* = 0,29). Diese Ergebnisse sind in Übereinstimmung mit vergleichbaren sprach- und länderübergreifenden Metaanalysen [8, 9, 41].

Die vorliegende Metaanalyse gibt zudem erstmals Hinweise darauf, dass sich DIGA von in Deutschland frei verfügbaren Interventionen in ihrer Wirksamkeit unterscheiden. Dieses Ergebnis ist angesichts der hohen Heterogenität der eingehenden Studien jedoch mit einer gewissen Unsicherheit behaftet und muss in künftigen Analysen weiter überprüft werden. Bei der Analyse der Follow-up-Daten zeigte sich in dieser Hinsicht kein signifikanter Unterschied mehr, wobei die Aussagekraft dieser Analyse durch die variablen Nachbeobachtungszeiträume (6 bis 32 Wochen) und die geringere Zahl der eingehenden Studien (*n* = 10) eingeschränkt ist.

Darüber hinaus zeigte sich eine große Bandbreite der Effektstärken der einzelnen Interventionen (*d* = 0,00–1,47). Mit Ausnahme von iFightDepression (95 %-KI: [−0,03; 0,41]) schienen alle Interventionen signifikant wirksamer in der Reduktion depressiver Symptomatik zu sein als die Kontrollbedingungen. Ein Urteil über Unter- oder Überlegenheit einzelner Interventionen ist basierend auf der hier berichteten Datenlage jedoch nicht möglich. Für die scheinbar am stärksten wirksame Intervention (selfapy Depression) liegt beispielsweise nur eine Studie vor, sodass es unsicher bleibt, ob dieser scheinbare Wirksamkeitsunterschied nicht besser durch Eigenschaften der Studie erklärt werden kann. Lediglich die Datenlage zu den Interventionen deprexis und moodgym stützen sich auf je mindestens 10 Studien, was eine gewisse Robustheit der hier gefundenen Effektstärken vermuten lässt. Diese Annahme wird gestützt durch die Beobachtung, dass das Ausmaß der Heterogenität in der Gesamtanalyse groß ist (*I*^*2*^ = 81 %), während sie bei den Studien zu deprexis und moodgym mit Heterogenitätsschätzern im mittleren Bereich (*I*^*2*^ = 51 % bzw. 55 %) liegt.

Die in der vorliegenden Arbeit gefundene mittlere Drop-out-Rate in den Interventionsgruppen von 29 % ist vergleichbar mit den Ergebnissen anderer Metaanalysen (z. B. [41]). Es lässt sich allerdings vermuten, dass im Routineeinsatz, außerhalb hochkontrollierter Studiensettings, die Drop-out-Raten höher ausfallen [42]. Eng mit der Frage der Adhärenz im Routineeinsatz ist auch die Frage der Ressourceneffizienz (also bspw. Kosten für die Gesundheitssysteme) der IBIs verbunden, zumal erste Befunde darauf hinweisen, dass eine höhere Adhärenz mit besseren Outcomes assoziiert ist [43]. Die uneinheitliche Forschungslage diesbezüglich verweist auf weiteren Forschungsbedarf [44, 45].

### Limitationen

Folgende Limitationen der vorliegenden Arbeit müssen bei der Interpretation der Ergebnisse berücksichtigt werden: Aufgrund der ausschließlichen Verwendung von Selbstbeurteilungsfragebögen als Hauptergebnismaß wies keine der eingeschlossenen Studien ein niedriges Biasrisiko auf. Über ein Drittel der Studien wies sogar ein hohes Biasrisiko auf. Dieses hohe Biasrisiko kam u. a. auch durch fehlende ITT-Analysen zustande. Sogenannte „Completer“-Analysen haben ein hohes Risiko einer Überschätzung der Effektstärke. Positiv ist hier anzumerken, dass unter den im DiGA-Verzeichnis zum Nachweis positiver Versorgungseffekte aufgeführten Studien [16, 22, 24, 25, 37–40] in der hier vorgenommenen Bewertung keine ein hohes Biasrisiko aufwies. Gleichwohl wurde auch bei Studien, auf deren Grundlage eine Aufnahme ins DiGA-Verzeichnis erfolgte, bereits auf problematische Qualitätsmängel hingewiesen und eine Verschärfung der methodischen Anforderungen angemahnt [46]. Ferner waren, wie bereits angemerkt, die Anzahl der Untersuchungen zu den einzelnen Interventionen sehr unausgeglichen.

Es zeigte sich zudem über die Studien hinweg eine große Bandbreite an unterschiedlichen Kontrollbedingungen, was einen erheblichen Einfluss auf die gefundenen Effektstärken gehabt haben könnte [47]. Beispielsweise wurde die einzige Intervention (ifighdepression), für die kein signifikanter Unterschied im Vergleich zur Kontrollbedingung gefunden werden konnte, nur in einer Studie untersucht und bei dieser Studie wurde eine aktive Kontrollbedingung gewählt (progressive Muskelrelaxation; [26]). Auch die vergleichsweise hohe Effektstärke in der Studie zur Intervention Selfapy könnte darauf zurückzuführen sein, dass die Kontrollgruppe in dieser Studie kaum Besserung zeigte [40]. Insbesondere die in vielen Studien gewählten Wartelistenkontrollgruppen sind möglicherweise sogar mit einem Noceboeffekt verbunden [48].

Hingewiesen muss auch darauf, dass bestimmte deutschsprachige Interventionen nicht in dieser Metaanalyse berücksichtigt wurden, obwohl sie in randomisierten Studien untersucht wurden, beispielsweise weil sie nur im Rahmen von Selektivverträgen verfügbar sind [49] oder nur für depressive Symptome im Rahmen bestimmter Erkrankungen (z. B. Diabetes [50]) untersucht wurden.

Schließlich ist zu bedenken, dass sich die Branche für IBIs sehr dynamisch entwickelt. So wurden beispielsweise allein in den wenigen Monaten seit dem Zeitpunkt der Datenerhebung für die vorliegende Metaanalyse drei weitere Interventionen mit der Indikation Depression vorläufig in das DiGA-Verzeichnis aufgenommen, und ihre Wirksamkeitsstudien dürften in naher Zukunft vorliegen. Dies verdeutlicht die Notwendigkeit einer kontinuierlichen Neubewertung der verfügbaren Evidenz.

## Fazit für die Praxis

- Unter Berücksichtigung der genannten Einschränkungen deuten die Ergebnisse dieser Analyse auf eine kurz- und langfristige Wirksamkeit der hier untersuchten in Deutschland verfügbaren IBIs für Depressionen hin. Es bleibt jedoch unklar, ob DiGA tatsächlich wirksamer sind als frei verfügbare Interventionen. Hier besteht weiterer Forschungsbedarf.
- Unsere Ergebnisse unterstützen die Empfehlung der aktuellen S3-Leitlinie zur Behandlung der unipolaren Depression. Diese sehen vor, dass IBIs bei leichten depressiven Episoden als Behandlung der ersten Wahl empfohlen werden. Dafür sprechen der niedrigschwellige Zugang und die im Vergleich zu Antidepressiva bessere Verträglichkeit. Bei mittelgradigen depressiven Episoden stellen IBIs eine Alternative und bei schweren depressiven Episoden eine mögliche Ergänzung zu Psychotherapie und Psychopharmakotherapie dar.

### Supplementary Information




