# Supplementary material for: Efficacy of internet-based interventions for depression available in Germany—A systematic review and meta-analysis
Source: Nervenarzt. 2024 Jan 23;95(3):206–15. [Article in German] doi: 10.1007/s00115-023-01587-0 (PMC10914865; doi:10.1007/s00115-023-01587-0)
Supplement: Supplementary file 1 [file 115_2023_1587_MOESM1_ESM.pdf]

Online-Zusatzmaterial zu

Wirksamkeit von in Deutschland verfügbaren internetbasierten Interventionen für Depressionen - ein systematisches Review mit Metaanalyse

Efficacy of Internet-based Interventions for depression available in Germany - a systematic review and meta-analysis

Raoul Haaf[1], Pia Vock[2], Nikolaj Wächtershäuser[2], Stephan Köhler[1], Christoph U. Corell[4-6], Jan Philipp Klein[2,3]

[1] *Klinik für Psychiatrie und Psychotherapie, Charité - Universitätsmedizin Berlin, Deutschland, corporate member der Freien Universität Berlin and Humboldt- Universität zu Berlin, Berlin, Deutschland*
[2]*Klinik für Psychiatrie, Psychosomatik und Psychotherapie, Universität zu Lübeck, Lübeck, Deutschland*
[3]*Center for Brain, Behavior and Metabolism, Universität zu Lübeck, Lübeck, Deutschland*
[4]*Klinik für Kinder- und Jugendpsychiatrie, Charité - Universitätsmedizin Berlin, Berlin, Deutschland*
[5]*Department of Psychiatry, The Zucker Hillside Hospital, Northwell Health, Glen Oaks, NY, USA*
[6]*Department of Psychiatry and Molecular Medicine, Donald and Barbara Zucker School of Medicine at Hofstra/Northwell, Hempstead, NY, USA*

1 Appendix

1.1 eMethodenteil

1.1.1 eTabelle 1: Übersicht über die Fragestellung der Studie gemäß PICO Schema

P – Population	Untersuchte Proband*innen sollten Personen ab einem Alter von 18 Jahren mit depressiver Symptomatik sein. Die Symptomatik musste mit validierten Fragebögen erhoben werden. Es fand kein Ausschluss aufgrund von Herkunft, Wohnort oder anderen soziodemografischen Variablen statt – speziell wurden auch Studien, die nicht in Deutschland durchgeführt wurden, eingeschlossen.
I – Intervention	Bei der Intervention musste es sich um eine internet-basierte Intervention im Sinne einer Selbstmanagementintervention handeln, die in Deutschland und auf Deutsch entweder frei verfügbar ist oder auf Rezept zu Lasten der Gesetzlichen Krankenversicherung verordnet werden kann (d.h. im DiGA-Verzeichnis gelistet ist). In dieser Intervention mussten evidenzbasierte psychotherapeutische Techniken angewendet werden und die vorgesehene Dauer der Intervention sollte mindestens zwei Wochen betragen. Der Behandlungsschwerpunkt der Intervention musste eine Depression sein, unabhängig von evtl. bestehenden komorbiden Störungen (nicht bspw. „Depression im Rahmen eines Diabetes“). Video- und Teletherapie sowie Begleitung durch Fachpersonal während der Intervention waren erlaubt, solang diese nur begleitend zur digitalen Selbstmanagementintervention waren und keine eigenständige Therapie darstellten. Bei mehrarmigen Studiendesigns wurde jene Experimentalgruppe ausgesucht, die der aktuell angebotenen Form der Intervention in der Versorgungsrealität am ehesten entspricht.
C – Control	Die Intervention musste mit einer randomisiert-kontrollierten Studie untersucht werden. Als Kontrollbedingungen durften folgende Methoden eingesetzt werden: Warteliste, Care as Usual (Regelbehandlung), Psychoedukation, Entspannung, Aufmerksamkeit,

	keine Behandlung. Spezifisch ausgeschlossen werden Studien, welche eine herkömmliche Psychotherapie in Person als Kontrollbedingung nutzten.
O – Outcome	Eingeschlossene Outcome-Maße beinhalteten validierte Selbst- und Fremdbeurteilungsbögen, welche Depressivität beurteilen (z.B. BDI-2, PHQ-9), oder Anzahl depressiver Symptome nach ICD-10 oder DSM (Auflage 4 oder 5).

1.1.2 eTabelle 2: Übersicht über die Schritte der Literaturrecherche

1 – Systematische Literaturrecherche in PubMed anhand des Searchstrings:	("Depression"[Mesh] OR "Depressive Disorder"[Mesh] OR "Dysthymic Disorder"[Mesh] OR "Depressive Disorder, Major"[Mesh] OR “depress*”[tiab] OR “dysthymi*”[tiab])<br>AND<br>("Internet-Based Intervention"[Mesh] OR "Telemedicine"[Mesh]) OR “online deliver*“[tiab] OR “online intervention*“[tiab] OR “online treat*“[tiab] OR “online therap*“[tiab] OR “online psychotherap*“[tiab] OR“digital intervention*“[tiab] OR “digital treat*“[tiab] OR “digital therap*“[tiab] OR “digital psychotherap*“[tiab] OR “mobile intervention*“[tiab] OR “mobile treat*“[tiab] OR “mobile therap*“[tiab] OR “mobile psychotherap*“[tiab] OR “mobile deliver* intervention*“[tiab] OR “mobile deliver* treat*“[tiab] OR “mobile deliver* therap*“[tiab] OR “mobile deliver* psychotherap*“[tiab] OR “mobile based intervention*“[tiab] OR “mobile based treat*“[tiab] OR “mobile based therap*“[tiab] OR “mobile based psychotherap*“[tiab] OR “internet intervention*“[tiab] OR “internet treat*“[tiab] OR “internet therap*“[tiab] OR “internet psychotherap*“[tiab] OR “internet deliver* intervention*“[tiab] OR “internet deliver* treat*“[tiab] OR “internet deliver* therap*“[tiab] OR “internet deliver* psychotherap*“[tiab] OR “internet based intervention*“[tiab] OR “internet based treat*“[tiab] OR “internet based therap*“[tiab] OR “internet based psychotherap*“[tiab] OR “self management intervention”[tiab] OR “web intervention*“[tiab] OR “web treat*“[tiab] OR “web therap*“[tiab] OR “web psychotherap*“[tiab] OR “web deliver* intervention*“[tiab] OR “web deliver* treat*“[tiab] OR “web deliver* therap*“[tiab] OR “web deliver* psychotherap*“[tiab] OR “web based intervention*“[tiab] OR “web based treat*“[tiab] OR “web based therap*“[tiab] OR “web based psychotherap*“[tiab])<br>AND<br>(“random*” OR “RCT”)<br>AND<br>(“German” OR “Germany”)
2 – Recherche auf Anbieterwebsites und im DiGA-Verzeichnis	Als zweite Domäne wurden aus den in Domäne 1 identifizierten Studien die Websites der Interventionshersteller nach zusätzlichen Studien zum Produkt durchsucht. Außerdem wurde das DiGA-Verzeichnis durchsucht nach: a) Interventionen, die den Kriterien entsprechen und noch nicht in Domäne 1 identifiziert wurden; sowie b) Studien zu Interventionen, die in Domäne 1 identifiziert wurden.

3 – Konsultation eines Experten	Im letzten Schritt wurde ein Experte für digitale Selbstmanagementinterventionen befragt (PK), ob es möglicherweise Interventionen gibt, die den Einschlusskriterien entsprechen aber nicht identifiziert wurden.

1.2 eErgebnisteil A

1.2.1 eTabelle 3: Charakteristika der eingeschlossenen Studien

Studie	Land	Intervention	Zielgruppe	Einschlusskriterien	N (IG; KG)	M Alter (SD)	%-weiblich	Baseline	Kontrolle	Primäres Ergebnismaß	Anleitung	Dauer	%-dropout (IG; KG)	Follow-up	Primär Ergebnisse (Cohen's d [95-%-KI])	Ergebnisse follow-up (Cohen's d [95-%-KI])	Kommentar
Beevers et al. (2017)	USA	deprexis	Allgemeinbevölkerung; 18-55 Jahre	QIDS-SR ≥ 10	376 (285; 91)	31,91 (11,2)	74	moderat	WL	QIDS-SR	nein	8	22; 11	nein	0,80 [0,56; 1,04]	-	
Beiwinkel et al. (2017)	DE	Novego	Allgemeinbevölkerung (Screening: frühere Depression (ICD), frühere Krankschreibung aufgrund einer Depression, aktuelle Krankschreibung); mind. 18 Jahre	PHQ-9 < 20; frühere Episode einer milden bis moderaten Depression oder Dysthymie (ICD)	180 (100; 80)	47,74 (10,92)	68	mild	WL; PE	BDI-II; (PHQ-9)	Ja, auf Anfrage via Mail oder Telefon (Psychologie B.Sc. oder höher)	12	56; 45	ja (12)	0,41 [0,12; 0,70]	0,19 [-0,22; 0,60]	
Berger et al. (2011)	DE; CH	deprexis	Allgemeinbevölkerung; mind. 18 Jahre	BDI-II > 13	51 (25; 26)*	38,80 (14,00)	70	schwer	WL	BDI-II	nein*	10	12; 15	ja (14), Follow-Up nur Vergleich zwischen begleitet und unbegleitet; für Kontrollgruppe keine Follow-Up Erhebung	0,66 [0,15; 1,17]	-	*Studie untersuchte neben einer nicht angeleiteten ebenfalls eine angeleitete Form der Intervention, welche jedoch nicht in die vorliegende Meta-Analyse einging.
Bruhns et al. (2021)	DE	COGITO	Studierende; mind. 18 Jahre	PHQ > 0	400 (200; 200)	22,99 (3,36)	89	moderat	WL	PHQ-9	nein	4	36; 33	nein	0,20 [0,02; 0,38]	-	
Bücker et al. (2018)	DE	deprexis	Personen mit pathologischem Spielautomaten-Spielverhalten und mit subjektiven Gefühlen von Traurigkeit und Verzweiflung; Alter 18-65 Jahre	selbstberichtete depressive Symptome (keine Angabe bezüglich eines Cut-Off Wertes)	140 (71; 69)	35,71 (10,21)	24	moderat	WL	PHQ-9	nein	8	56; 39	nein	0,75 [0,42; 1,08]	-	
Ellis et al. (2011)	AUS	moodgym	Studierende; 18-25 Jahre	K10 (Kessler Psychological Distress Scale) - niedriger bis moderater psychologischer Distress (keine Angabe bezüglich genauer Cut-Off Werte)	26 (13; 13)*	19,67 (1,66)	77	mild	TAU	DASS-21	nein	3	0; 0	nein	0,40 [-0,33; 1,13]	-	* Studie untersuchte außerdem eine Online-Selbsthilfegruppe (MoodGarden), welche jedoch nicht in die vorliegende Meta-Analyse einging.
Farrer et al. (2011)	AUS	moodgym	Allgemeinbevölkerung (Anrufer bei Krisenhotline); mind. 18 Jahre	K10 > 22 (Kessler Psychological Distress Scale)	73 (38; 35)*	41,53 (12,24)	82	schwer	TAU	CES-D	nein*	6	29; 23	ja (18)	0,76 [0,21; 1,31]	1,19 [0,54; 1,84] (web only)	*Studie untersuchte neben einer nicht angeleiteten ebenfalls eine angeleitete Form der Intervention, welche jedoch nicht in die vorliegende Meta-Analyse einging.
Fischer et al. (2015)	DE	deprexis	Personen mit einer multiplen Sklerosis Diagnose und depressiven Symptomen (rektrutiert über Datenbank der MS-Ambulanz des UKE und Internetforen); 18-65 Jahre	selbstberichtete depressive Symptome (keine Angabe bezüglich eines Cut-Off Wertes)	90 (45; 45)	45,28 (11,6)	78	mild	WL	BDI-II	nein	9	22; 20	ja (24), fehlende Daten (es ist nur der BDI Mittelwert für die Interventionsgruppe vor und nach der Intervention berichtet)	0,53 [0,10; 0,96]	-	
Fuhr et al. (2018)	DE	deprexis	Personen, die aufgrund einer depressiven Symptomatik auf der Warteliste für einen ambulanten Psychotherapieplatz	depressive Symptome (MINI; keine Angaben zu Cut-Off Werten)	31 (17; 14)	37.70 (13,64)	67	moderat	WL (PT)	PHQ-9	nein	10	18; 7	nein	-0,04 [-0,67; 0,59]	-	

			der Hochschulambulanz Tübingen standen; mind. 18 Jahre														
Gilbody et al. (2015)	UK	moodgym	primäre Versorgung (aktuell nicht in Psychotherapie); mind. 18 Jahre	PHQ-9 ≥ 10	481 (242; 239)*	39,86 (12,65)	67	moderat - schwer	TAU	PHQ-9	Ja (wöchentliche Telefonate)	16	25; 25	ja (32; 80)	0,01 [-0,24; 0,26]	0,32 [0,05; 0,59] (32 Wochen)	*Studie untersuchte außerdem eine dritte Gruppe mit der in Deutschland nicht verfügbaren Anwendung (“Beating the Blues”), welche nicht in die vorliegende Meta-Analyse einging.
Gräfe et al. (2020)	DE	deprexis	Patient:innen mit milder bis moderater depressiven Episode nach ICD; Allgemeinbevölkerung (rekrutiert über Krankenkasse); mind. 18 Jahre	PHQ-9 > 4	3805 (1904; 1901)	45 (10,90)	79	moderat	TAU; PE	PHQ-9	nein	12	27; 26	ja (12; 36)	0,37 [0,29; 0,45]	0,23 [0,15; 0,31] (12 Wochen)	
Høifødt et al. (2013)	NOR	moodgym	Allgemeinbevölkerung / primary care (rekrutiert durch Hausarztpraxen, Hausarzt-Pflegepersonal, Warteliste von psychiatrischer ambulanter Klinik); 18-65 Jahre	BDI-II 10-40	106 (52; 54)	36,10 (11,30)	73	moderat	WL	BDI-II	Ja, kurzer therapeutischer face to face Kontakt und personalisierte E-Mails zwischen den Sessions	6	29; 13	ja (18), jedoch falscher Vergleich (die Kontrollgruppe hat während des Follow-Up Zeitraums die Intervention erhalten. Somit haben beim Follow-Up Zeitpunkt beide Gruppen die Intervention erhalten (nur eben verzögert))	0,65 [0,18; 1,12]	-	
Klein et al. (2016)	DE	deprexis	Patient:innen mit milder bis moderater depressiven Symptomatik (Selbstbericht); Allgemeinbevölkerung und stationäres sowie ambulantes klinisches Setting; 18-65 Jahre	PHQ-9 5-14	1013 (509; 504)	42,85 (11,00)	69	moderat	WL	PHQ-9	ja/nein, Proband:innen mit einem Baseline PHQ-9 zwischen 10-14 erhielten begleitete Version in Form von wöchentlichem E-Mail Support	12	22; 21	ja (12)	0,39 [0,14; 0,64]	0,32 [0,07; 0,57]	
Krämer et al. (2022)	DE	Selfapy	Allgemeinbevölkerung; 18 - 65 Jahre	BDI-II ≥ 13; Diagnose einer Major Depression / Dysthymie basierend auf dem MINI	250 (150; 100)*	37,00 (11,00)	83	schwer	WL; AC	BDI-II	nein*	12	23; 47	ja (12), jedoch nur within-group Daten für den Follow-Up Erhebungszeitpunkt, daher nicht teil der Meta-analyse	1,47 [1,22; 1,72]	-	*Studie untersuchte neben einer nicht angeleiteten ebenfalls eine angeleitete Form der Intervention, welche jedoch nicht in die vorliegende Meta-Analyse einging.
Löbner et al. (2018)	DE	moodgym	Allgemeinbevölkerung (rekrutiert aus Hausärzt:in-Setting); mind. 18 Jahre	Diagnose einer aktuellen milden oder moderatoren ersten oder wiederkehrenden depressiven Episode nach ICD-10; PHQ-9 5-19	647 (320; 327)	43,89 (13,26)	68	moderat	TAU	BDI-II; (PHQ-9)	nein	6	19; 6	ja (18)	0,36 [0,19; 0,53]	0,41 [0,21; 0,61]	
Lüdtke et al. (2018)	DE	COGITO	Allgemeinbevölkerung (rekrutiert über ambulante Versorgung, Online-Foren für Depressionen); 18-65 Jahre	subjektives Bedürfnis nach einer Behandlung um depressive Symptome zu reduzieren	88 (44; 44)	42,89 (11,28)	78	moderat	WL	PHQ-9	nein	4	20; 11	nein	0,00 [-0,41; 0,41]	-	
Mackinnon et al. (2008)	AUS	moodgym	Allgemeinbevölkerung; 18-52 Jahre	keine Angabe	360 (182; 178)*	k.A.	k.A.	moderat	AUF	CES-D	Ja, wöchentliche 10 minütige Telefonate	6	25; 11	ja (18, 42)	0,41 [0,19; 0,63]	0,22 [0,02; 0,42] (18 Wochen)	*Studie untersuchte zudem einen Interventionsarm mit Teilnehmer*innen die Zugang zu einer Depressionsinformationsseite erhielten, welcher jedoch nicht in die vorliegende Meta-Analyse einging.

Meyer et al. (2009)	DE	deprexis	Allgemeinbevölkerung (rekrutiert über Internetforen); mind. 18 Jahre	keine Angabe	396 (320; 76)	34,76 (11,60)	76	moderat	WL	BDI-I	nein	9	50; 25	ja (9; 24)	0,30 [0,05; 0,55]	0,26 [-0,11; 0,63] (9 Wochen)	
Meyer et al. (2015)	DE	deprexis	Patient:innen mit moderater bis schwerer oder schwerer depressiven Symptomatik (Selbstbericht); Allgemeinbevölkerung und stationäres sowie ambulantes klinisches Setting; 18-65 Jahre	PHQ-9 > 14	163 (78; 85)	42,00 (11,39)	75	moderat - schwer	WL	PHQ-9	nein	12	22; 14	ja (12)	0,57 [0,22; 0,92]	0,33 [-0,02; 0,68]	
Miegel et al. (2019)	DE	Novego	Allgemeinbevölkerung; 18-75 Jahre	depressive Symptome (keine Diagnose erforderlich, sondern auf Basis der subjektiven Einschätzung der Teilnehmer:innen; außerdem subjektive Schmerzsymptome),	142 (71; 71)	48,15 (11,87)	77	moderat	WL	BDI-II	Ja, Möglichkeit bei Fragen ein Team aus Psycholog:innen zu kontaktieren	12	32; 15	nein	0,42 [0,09; 0,75]	-	
Moritz et al. (2012)	DE	deprexis	Allgemeinbevölkerung (rekrutiert über Support Networks im Internet für Depressionen); 18-65 Jahre	keine Angabe	210 (105; 105)	38,57 (13,29)	79	schwer	WL	BDI-I	nein	8	24; 14	nein	0,36 [0,03; 0,69]	-	
Moritz et al. (2016)	DE	Novego	Personen mit einer Schizophreniediagnose (ehemalige Patient:innen einer Klinik für Psychiatrie und Psychotherapie (UKE), modertierte Internetforen für Schizophrenie); 18-65 Jahre	aktuell subjektiv erlebte depressive Symptome; kein formaler Cut-Off Wert	58 (31; 27)	40,62 (10,22)	53	schwer	WL	CES-D	nein	12	19; 11	nein	0,91 [0,36; 1,46]	-	
Oehler et al. (2020)	DE	iFightDepression	Allgemeinbevölkerung; mind. 18 Jahre	Diagnose einer aktuellen milden oder moderatoren depressiven Störung oder Dysthymie basierend auf dem MINI; PHQ-9 5-14	347 (173; 174)	42,30 (12,40)	79	moderat	PMR	IDS-SR	Ja, 5 telefonische Termine mit Psychotherapeut:innen	6	18; 26	ja (12; 24; 48)	0,19 [-0,03; 0,41]	0,28 [0,06; 0,50] (12 Wochen)	
Philipps et al. (2014)	UK	moodgym	Arbeitnehmer:innen; mind. 18 Jahre	PHQ-9 Score von 2 oder mehr auf 5 der 9 Items, einschließlich ein Score von 2 oder mehr auf Item 1 (wenig Interesse an Aktivitäten) oder Item 2 (sich hoffnungslos fühlen); Proband:innen müssen zusätzlich bestätigen, dass zumindest eins der Items ein Problem für sie darstellt, welches es für sie schwierig macht zu arbeiten, den Haushalt oder zwischenmenschliche Kontakte zu pflegen	637 (318; 319)	42,45 (9,60)	51	moderat	PE	PHQ-9	Nein, KG und EG erhielten wöchentliche 10 minütige telefonische Interviews - Zweck: Teilnahmemotivation erhöhen, Risikoscreening, Sammlung von Nutzungsdaten	5	46; 41	ja (7), jedoch fehlende Daten (kombinierte Effektstärke aus post intervention und follow up)	0,07 [-0,09; 0,23]	-	
Powell et al. (2013)	UK	moodgym	Allgemeinbevölkerung; mind. 18 Jahre	keine Angabe	3070 (1534; 1536)	41,14 (13,01)	78	k.A.	WL	WEMWBS	nein	6	64; 21	ja (6), jedoch fehlende Daten	0,21 [0,11; 0,31]	-	

Schröder et al. (2014)	GER	deprexis	Personen mit einer Epilepsie-Diagnose (rekrutiert über Datenbank eines Epilepsiezentrums und Internetforen); mind. 18 Jahre	selbstberichtete depressive Symptome	78 (38; 40)	37,59 (10,94)	76	moderat	WL	BDI-I	nein	9	34; 20	nein	0,29 [-0,22; 0,80]	-	
Sethi (2013)	AUS	moodgym	Allgemeinbevölkerung (Jugendzentren und Studierende); 18-25 Jahre	DASS-21: 10-20 (Depression) und / oder 8-14 (Angst)	46 (23; 23)*	20,08 (1,72)	67	moderat	TAU	DASS-21	Ja, Psycholog:in steht zur Verfügung bei aufkommenden Fragen	5	0; 0	nein	0,70 [-0,10; 1,50]	-	*Studie untersuchte außerdem eine Gruppe mit face-to-face Therapie und eine mit blended Treatment, welche beide nicht in die vorliegende Meta-Analyse miteingingen
Twomey et al. (2014)	IRE	moodgym	Personen auf der Warteliste für öffentliche mental health services und depressiven, ängstlichen und / oder Stress-Symptomen; Alter 18-61 Jahre	depressive, ängstliche und / oder Stresssymptome, nachgewiesen durch Überweisungsschein	149 (80; 69)	35,30 (10,30)	74	moderat	WL	DASS-21	nein	5	56; 29	Nein, bzw. zu wenig Daten	0,19 [-0,30; 0,68]	-	

Anmerkung. %-weiblich = Prozentanteil weiblicher Probandinnen; AC = Achtsamkeit; AUF = Aufmerksamkeit, Baseline = Schwere der depressiven Symptomatik vor Untersuchungszeitraum; Dauer = Interventionsdauer in Wochen; Follow-up = Nachverfolgungszeitraum nach Interventionsende in Wochen; IG = Interventionsgruppe; KG = Kontrollgruppe; PE = Psychoedukation; PMR = Progressive Muskelrelaxation; TAU = Regelversorgung; WL = Warteliste; WL (PT) = Warteliste für ambulante Psychotherapie; nein = Studie untersuchte neben einer nicht angeleiteten ebenfalls eine angeleitete Form der Intervention, welche jedoch nicht in die vorliegende Meta-Analyse einging*

1.2.2 *eAbbildung 1: Flussdiagramm zum Identifikationsprozess relevanter Studien*

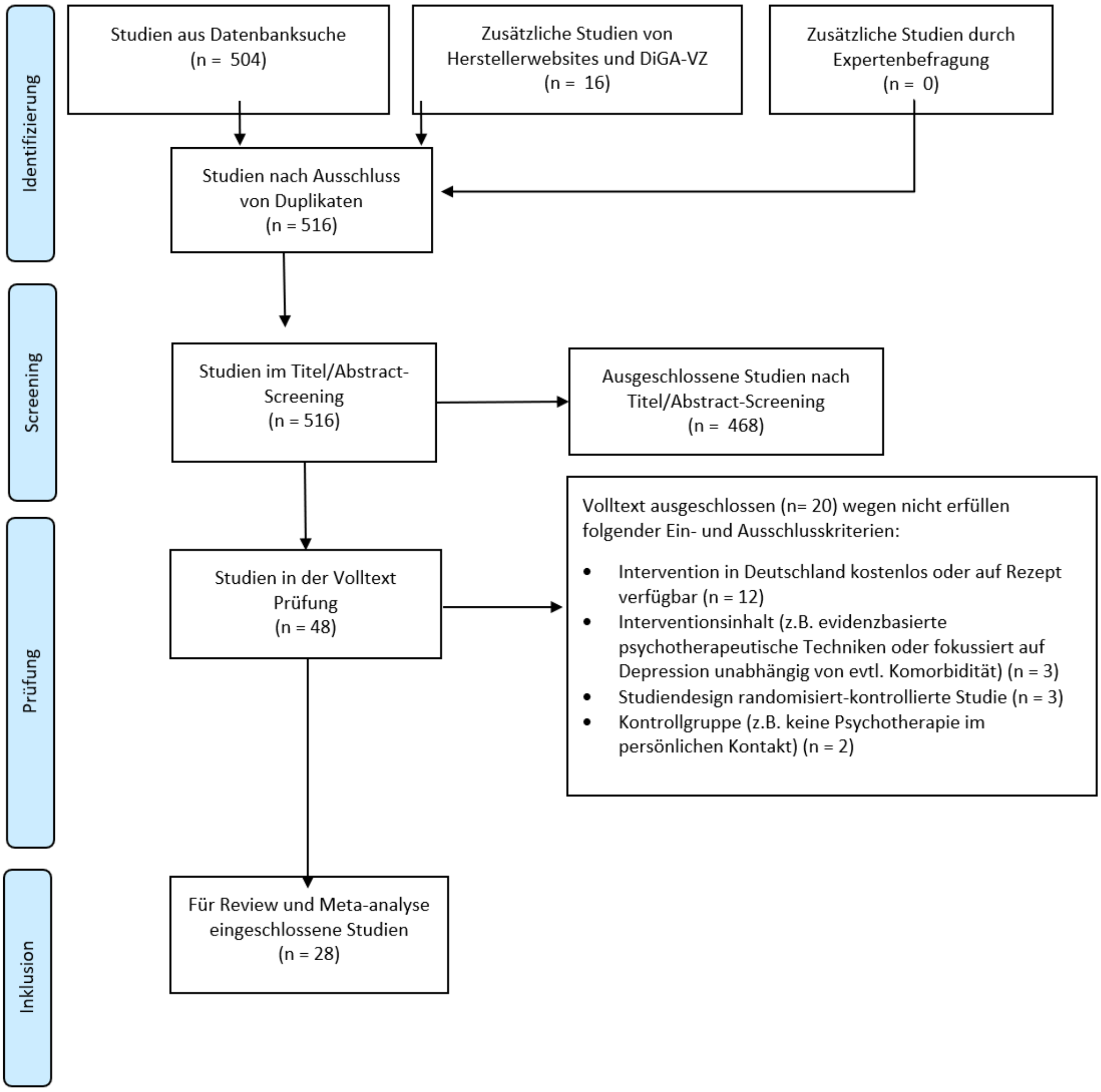

1.2.3 *Risk of Bias*

Alle im Review eingeschlossenen Studien wurden hinsichtlich ihres Risk of Bias unabhängig von zwei Reviewern (NW und PV) bewertet. Dies wurde anhand des Risk of Bias 2 (RoB-2) Tools der Cochrane-Collaboration auf Studienebene und Domänenebene durchgeführt. Das Tool enthält folgende Domänen: (1) Bias durch den Randomisierungsprozess, (2) Bias durch Abweichung von der geplanten Intervention, (3) Bias durch fehlende Daten, (4) Bias durch die Messung des Outcomes, (5) Bias durch die Auswahl des berichteten Ergebnisses, sowie ein sich aus den Domänenratings ergebendes Gesamtrating. Pro Domäne beinhaltet das Tool Fragen zur Methodik der zu bewertenden Studie sowie einen Entscheidungsbaum. Dadurch können die Domänen dreistufig klassifiziert werden in a)

niedriges Bias-Risiko, b) mittelgroßes Bias-Risiko und c) hohes Bias-Risiko. Abweichungen in der Bewertung zwischen den beiden Reviewern wurden im Anschluss identifiziert und Unstimmigkeiten durch Konsens geklärt.

eAbbildung 2: Zusammengefasstes Bias-Risiko der eingeschlossenen Studien auf den Domänen des RoB-2 Tools.

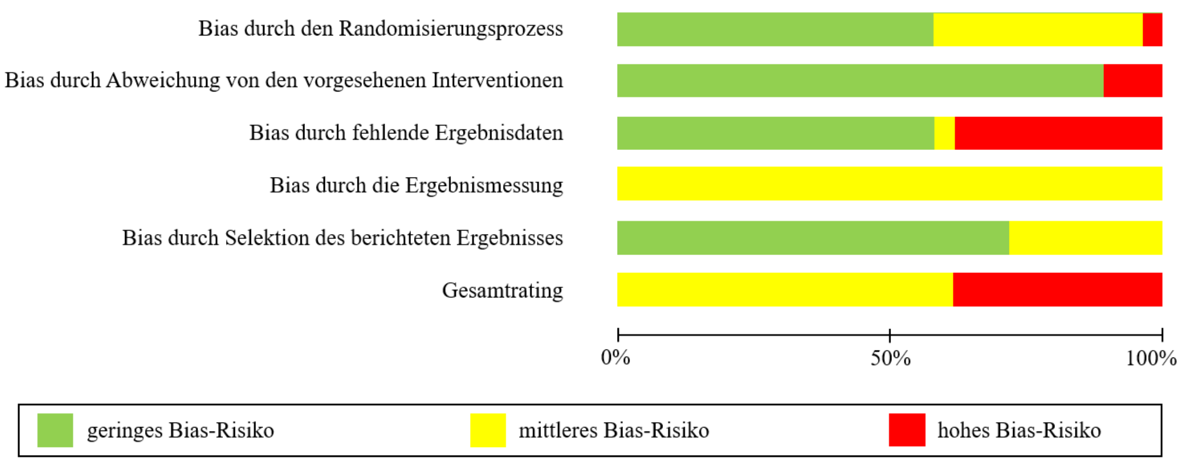

eAbbildung 3: Bias-Risiko auf Studienebene auf den Domänen des RoB-2 Tools.

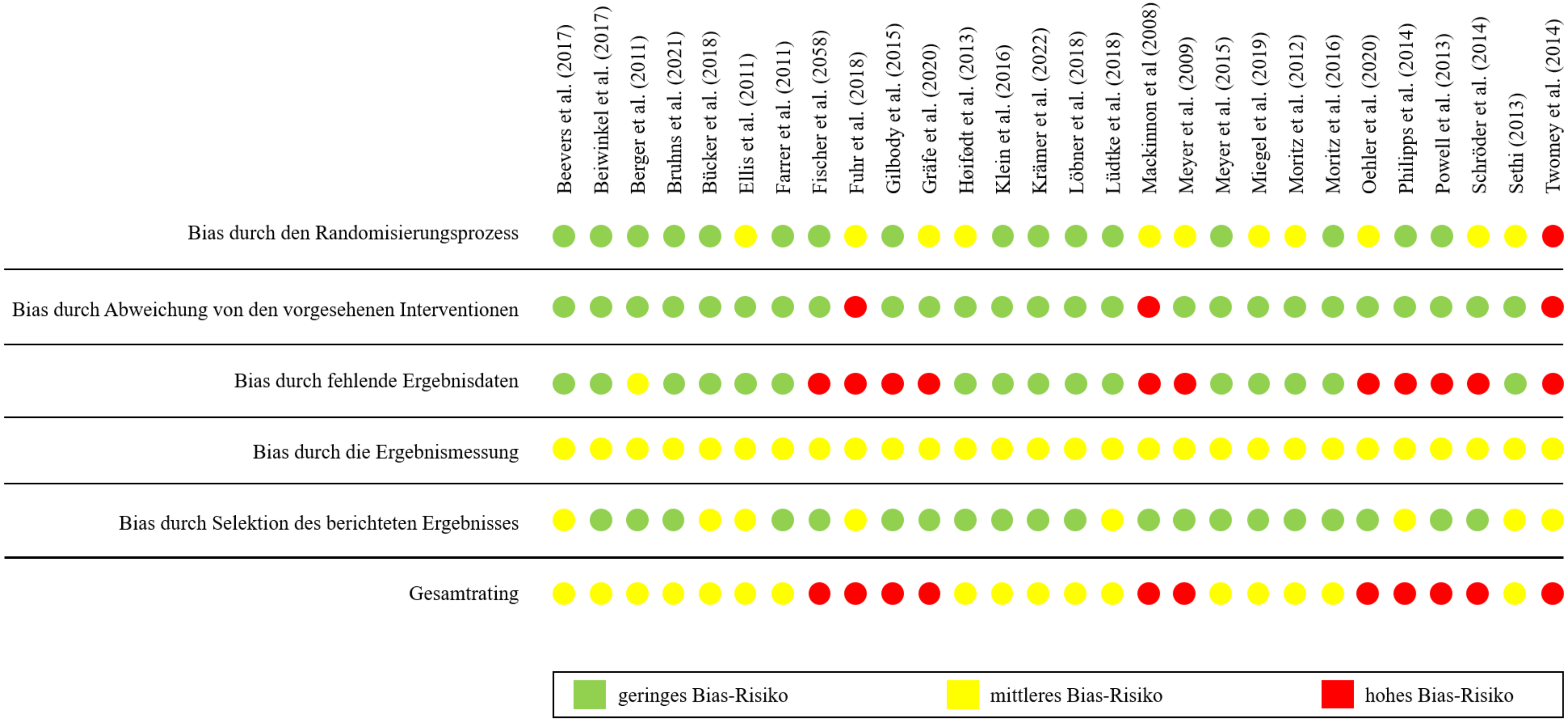

1.2.4 *Follow-up Analyse*

Von den 16 identifizierten Studien, die zusätzlich zum Post-Interventions-Zeitpunkt eine Follow-Up Erhebung der depressiven Symptomatik durchführten, konnten sechs Studien aufgrund fehlender oder unpassender Daten nicht in die Analyse eingeschlossen werden: Berger et al. (2011), Fischer et al. (2015) und Krämer et al. (2022) berichteten die Daten für die Follow-Up Erhebung nur für die Interventionsgruppe und nicht für die Kontrollgruppe. In der Studie von Høifødt et al. (2013) erhielt die Kontrollgruppe zum Zeitpunkt der Follow-Up Erhebung die Intervention bereits. Phillips et al. (2014) gibt die Effektstärke nur in Form einer kombinierten Effektstärke aus dem Postintervention und Follow-Up Erhebungszeitpunkt an und Powell et al. (2013) berichtet zu wenig Daten, um die Effektstärke nach Cohen'ʻs *d* zu berechnen.

1.2.5 *Sensitivitätsanalysen*

1) hohes Bias Risiko. Insgesamt zeigten 11 der 28 inkludierten Studien ein hohes Bias-Risiko. In einer separaten Sensitivitätsanalyse, in der die elf Studien ausgeschlossen wurden, die in der Gesamtbeurteilung ein hohes Bias-Risiko erhielten, zeigte sich eine Effektstärke von d = 0,57, 95-%-KI: [0,38; 0,76], I^2 = 82 % zum Post-Interventions-Zeitpunkt. Die Analyse der Follow-Up Daten ergab nach Ausschluss von fünf Studien mit einer hohen Bias-Risiko-Beurteilung eine Effektstärke von d = 0,40, 95-%-KI: [0,20; 0,59], I^2 = 45 %.

2) aktive Kontrollgruppe. Für den Postinterventions-Zeitpunkt zeigte sich nach Ausschluss von sechs Studien mit einer aktiven Kontrollgruppe ein kleiner Effekt von d = 0,40, 95-%-KI: [0,29; 0,51], I^2 = 61 %. Die Analyse der Follow-Up Daten ergab nach Ausschluss von insgesamt vier Studien mit einer aktiven Kontrollgruppe eine Effektstärke von d = 0,38, 95-%-KI: [0,23; 0,53], I^2 = 28 %.

3) Population mit einer zusätzlichen komorbiden psychischen oder neurologischen Störung. In einer weiteren Sensitivitätsanalyse wurden Studien ausgeschlossen, die eine spezifische Stichprobe mit einer zusätzlichen neurologischen oder psychischen Erkrankung untersuchten (pathologisches Glücksspielverhalten (18) , multiple Sklerose (19), Schizophrenie (39), Epilepsie (17). Nach Ausschluss dieser vier Studien zeigte sich eine Effektstärke von d = 0,40, 95-%-KI: [0,28; 0,52], I^2 = 83 %) zum Post-Interventions-Zeitpunkt.

4) nicht- deutschsprachige Studien. Da einige der Interventionen in mehreren Sprachen verfügbar sind und sich die Interventionen auf den verschiedenen Sprachen unterscheiden könnten, wurde eine Sensitivitätsanalyse hinsichtlich der Studienherkunft durchgeführt. Schließt man die Studien an nicht-deutschsprachigen Stichproben (n = 10) aus, so erhält man eine Effektstärke von d = 0,46, 95-%-KI: [0,32; 0,61].

1.2.6 *Subgruppenanalyse*

eTabelle 4: Übersicht über die durchgeführte Subgruppenanalyse zum Faktor DiGA oder frei verfügbar

Faktor	n	d	95-%-KI	I^2
**DiGA (ja/nein)**				
DiGA	15	0.56*	[0.38, 0.74]	83%
freiverfügbar	13	0.24*	[0.14, 0.33]	44%
DiGA (FU)	5	0.24	[0.17, 0.31]	0%
freiverfügbar (FU)	5	0.36	[0.19, 0.53]	55%

*Anmerkung: FU = Follow-Up-Zeitpunkt; n = Studienanzahl; d = Effektgröße (Cohen's d); 95-%-KI = 95%-Konfidenzintervall; I^2 = Maß für die vorliegende Heterogenität nach Higgins; * = statistisch signifikanter Unterschied zwischen den beiden Subgruppen (p < 0.05).*

1.3 eErgebnisteil B: Tabellarische und narrative Übersicht über die identifizierten Interventionen

Der folgende Überblick ergänzt eine tabellarische Übersicht, die von drei der Autor:innen (JPK, PV, NW) bereits online zur Verfügung gestellt wurde (DOI:10.13140/RG.2.2.31734.52803).

1.3.1 COGITO

Die Anwendung *COGITO* wurde am Universitätsklinikum Hamburg Eppendorf entwickelt. COGITO ist nicht im DiGA-Verzeichnis gelistet und kann kostenlos genutzt werden. Es handelt sich um eine Smartphone-App, welche für die mobilen Betriebssysteme iOS und Android zur Verfügung steht. Das für depressive Störungen relevante Basispaket „Stimmung und Selbstwert" des Kurses besteht aus 66 kurzen Übungen, welche auf vier Module aufgeteilt sind: Lebensfreude & Neue Blickwinkel (kognitive Strategien), Aktivität & Energie (Verhaltensaktivierung, Werte, Ressourcen), Kommunikation & Beziehungen (soziale Kompetenzen), und Achtsamkeit & Innere Ruhe (Akzeptanz, Dankbarkeit, Entspannung). Zusätzlich können eigene Übungen erstellt werden. Der therapeutische Ansatz des Kurses basiert vor allem auf dem Metakognitiven Training, der kognitiven Verhaltenstherapie und dritte-Welle Verfahren wie Achtsamkeit und Akzeptanz. Die App wird ohne Begleitung durch Fachpersonal benutzt. Die einzelnen Übungen nehmen jeweils nur wenige Minuten in Anspruch und sollen somit gut in den Alltag integrierbar sein. Es gibt keine empfohlene Nutzungsdauer, allerdings untersuchten beide identifizierten RCTs die Effektivität von COGITO über einen Interventionszeitraum von vier Wochen (13, 14). Um die Motivation und die Adhärenz zu fördern, kommen Gamification-Elemente in Form von Medaillenauszeichnung zum Einsatz, ebenso wie automatische wöchentliche Rückmeldungen mit einem Überblick der Anzahl an absolvierten Übungen in den letzten sieben Tagen. COGITO wurde bisher in zwei randomisiert kontrollierten Studien untersucht (13, 14). Beide Studien gingen in die vorliegende Metaanalyse ein.

**COGITO**[4]	
Indikation	Depressive Störung
Therapieprinzip	KVT, Metakognitives Training (MCT), Achtsamkeit und Akzeptanz
Wirkstärke im Vergleich zur Kontrollgruppe	RCTs: d = 0,26 (PP) (400 Teilnehmende) (Bruhns et al., 2021); d = 0,00 (ITT) (90 Teilnehmende) (Lüdtke et al., 2018)
Möglichkeit der Integration in die f2f-Behandlung	0: Intervention kann unabhängig von der f2f-Behandlung bearbeitet werden
PZN	–
Sprachen	Deutsch, Englisch, Türkisch, Arabisch, Serbisch, Norwegisch, Italienisch, Spanisch, Portugiesisch, Französisch, Finnisch, Polnisch, Persisch

Plattformen	Apple App Store, Google Play Store

[4]Im iOS App-Store und Google Play Store zum Download kostenlos verfügbar / weitere Informationen unter https://clinical-neuropsychology.de/cogito/

1.3.2 deprexis

Die Intervention *deprexis* der GAIA AG ist seit Frühjahr 2021 dauerhaft im DiGA-Verzeichnis gelistet. deprexis ist bei Vorliegen einer leichten bis schwer ausgeprägten depressiven Störung (F32.0, F32.1, F32.2, F33.0, F33.1, F33.2) indiziert und kann ab einem Alter von 18 Jahren verordnet werden. Der therapeutische Ansatz des Kurses basiert größtenteils auf Techniken der kognitiven Verhaltenstherapie. Insgesamt besteht der Kurs aus zwölf Modulen, wovon neben einem Einleitungs- und einem Abschlussmodul zehn Module psychotherapeutischen Inhalte vermitteln. Dabei gibt es folgende inhaltliche Bausteine: Verhaltensaktivierung, Kognitive Umstrukturierung, Achtsamkeit und Akzeptanz, Interpersonelle Fähigkeiten, Relaxation und Lifestyle-Modifikation, Problemlösen, Kindheitserfahrungen und Schemata, Positive Psychologie, Emotionsfokussierte Techniken, Psychoedukation. Die Module bestehen aus Informationstexten, Video- und Audiomaterial sowie simulierten Dialogen, welche dynamisch auf User-Input reagieren sollen (23). Pro Modul wird eine Bearbeitungsdauer von 10 bis 60 Minuten geschätzt. Der Kurs ist nicht durch Fachpersonal begleitet. Die gesamte Nutzungsdauer beträgt 90 Tage. Die Wirksamkeit von deprexis wurde bisher in 13 randomisiert kontrollierten Studien nachgewiesen. Zwölf dieser Studien wurden in einer deprexis spezifischen Metaanalyse ausgewertet (Twomey et al. 2020). In die vorliegende Metaanalyse gingen elf dieser Studien ein.

**deprexis**[3]	
Indikation	Depressive Störung (leicht bis schwer)
Therapieprinzip	KVT, aber auch andere psychotherapeutische Techniken; alle Inhalte werden in simulierten Dialogen vermittelt
Wirkstärke im Vergleich zur Kontrollgruppe	Metaanalyse: g = 0,51 (12 Studien mit 2901 Teilnehmenden) (Twomey et al., 2020)
PZN	17265872
Möglichkeit der Integration in die f2f-Behandlung	0: DiGA kann unabhängig von der f2f-Behandlung bearbeitet werden
Sprachen	Chinesisch, Deutsch, Englisch, Französisch, Griechisch, Italienisch, Portugiesisch, Schwedisch, Spanisch
Plattformen	Webanwendung

[3]Kontakt: info@deprexis.de

1.3.3 iFightDepression

Der iFightDepression-Kurs wurde von der European Alliance Against Depression entwickelt und richtet sich an Erwachsene und Jugendliche ab 15 Jahren, die an einer leichten bis mittelgradigen depressiven Störung leiden. Der Kurs muss durch einen Arzt oder Psychotherapeuten, der vorher

eine kostenlose spezifische Schulung durchlaufen hat, freigeschaltet werden. Die Nutzung ist dann für beide Seiten kostenlos. So kann iFightDepression nur in Begleitung durch therapeutisches Personal genutzt werden. Dabei bestehe die Begleitung vor allem daraus, den Patient*innen administrative und motivationale Unterstützung zu geben. Der therapeutische Ansatz des Kurses basiert auf Techniken der kognitiven Verhaltenstherapie und besteht aus sechs Modulen: Denken, Fühlen und Tun (Psychoedukation, Selbstbeobachtung), Schlaf und Depression, Planen und Durchführen angenehmer Aktivitäten (Aktivitätenaufbau), Dinge schaffen (Problemlösetraining, Zielsetzung), Negative Gedanken identifizieren, Negative Gedanken ändern (kognitive Umstrukturierung). Die Module bestehen aus Informationstexten, Arbeitsblättern und Aufgaben. Es gibt keine empfohlene Nutzungsdauer, allerdings untersuchte das identifizierte RCT die Effektivität vom iFightDepression-Kurs über einen Interventionszeitraum von sechs Wochen. Eine durchschnittliche Bearbeitungsdauer pro Modul ist nicht angegeben. Die Wirksamkeit von iFightDepression bei einer leichten und mittelgradigen depressiven Störung wurde in einer Studie untersucht, die in die vorliegende Metaanalyse aufgenommen wurde.

**iFightDepression**[5]	
Indikation	Depressive Störung
Therapieprinzip	KVT, u.a. kognitive Techniken, Verhaltensaktivierung, Schlafregulation
Wirkstärke im Vergleich zur Kontrollgruppe	RCT: d = 0,19 (ITT) (Follow-up nach 3 Monaten: d = 0,28) (348 Teilnehmende) (Oehler et al., 2020)
Möglichkeit der Integration in die f2f-Behandlung	3: Intervention kann nicht unabhängig von f2f-Behandlung verwendet werden, Integration in f2f-Behandlung notwendig
PZN	–
Sprachen	Deutsch, Englisch, Spanisch, Französisch, Niederländisch, Italienisch, Portugiesisch, Russisch, Polnisch, Norwegisch, Türkisch, Griechisch, Estnisch, Bulgarisch, Katalanisch, Ungarisch, Albanisch
Plattformen	Webanwendung

[5] Freier Zugang für Patient*innen, deren Behandler*in ein 70-minütiges Webinar abgeschlossen hat: https://ifightdepression.com/webinar/

1.3.4 moodgym

Moodgym wurde von der Australian National University entwickelt und ist seit 2016 auf Deutsch frei im Internet verfügbar. Die Webanwendung moodgym wurde ursprünglich von Wissenschaftler*innen der Australian National University in englischer Sprache entwickelt. Es gibt keine offizielle Indikation für die Nutzung, allerdings zielt moodgym primär auf die Verbesserung von depressiver Symptomatik, sekundär auf Ängstlichkeit und generelles psychisches Wohlbefinden ab. Es gibt keine empfohlene Nutzungsdauer, die meisten Studien nutzten jedoch einen Interventionszeitraum von sechs Wochen. Insgesamt besteht moodgym aus sieben Modulen, davon fünf mit inhaltlichen

Schwerpunkten und je eines zur Einleitung und zum Review. Die Bearbeitungszeit wird pro Modul auf 30 bis 60 Minuten geschätzt. Die inhaltlichen Schwerpunkte der Module sind: Emotionen und Gedanken sowie kognitive Verzerrungen, Distanz zu automatischen Gedanken schaffen, Stressabbau durch Relaxation und Identifikation von Stressoren, sowie soziale Beziehungen und Problemlösefähigkeiten. Die Module bestehen aus Informationstexten, Animationen sowie interaktiven Übungen und Quizzen. Der therapeutische Ansatz des Kurses basiert auf Techniken der kognitiven Verhaltenstherapie. Der Kurs ist unbegleitet. Moodgym wurde in mehreren randomisiert kontrollierten Studien sowie in einer Metaanalyse, die elf Studien einschloss, untersucht (Twomey et al. 2017). Eine Studie überprüfte explizit die Wirksamkeit der deutschen Version von moodgym (31). In die vorliegende Metaanalyse gingen insgesamt zehn dieser Studien ein.

**Moodgym**[6]	
Indikation	Depressive Störung
Therapieprinzip	KVT, v.a. kognitive Techniken aber auch andere Themen wie Aktivierung und Beziehungen
Wirkstärke im Vergleich zur Kontrollgruppe	RCT der deutschen Version: d = 0,36 (ITT) (647 Teilnehmende) (Löbner et al., 2018); Metaanalyse der englischen Version: g = 0,36 (11 Studien mit 5745 Teilnehmenden) (Twomey & O'Reilly, 2017)
Möglichkeit der Integration in die f2f-Behandlung	1: Intervention kann unabhängig von f2f-Behandlung bearbeitet oder in f2f-Behandlung integriert werden
PZN	_
Sprachen	Deutsch, Englisch
Plattformen	Webanwendung

[6] Freier Zugang für alle: https://moodgym.de/

1.3.5 Novego: Depressionen bewältigen

Das Programm Novego: Depressionen bewältigen der IVPNetworks GmbH wurde am 10. Oktober 2021 vorläufig in das DiGA-Verzeichnis aufgenommen. Es handelt sich um eine deutschsprachige Web-Anwendung, die bei Vorliegen einer leichten bis mittelgradigen depressiven Störung (ICD-10: F32.0, F32.1, F33.0, F33.1) sowie Dysthymie (F34.1) indiziert ist und ab einem Alter von 18 Jahren verordnet werden kann. Die Inhalte des Programms basieren auf den Methoden der kognitiven Verhaltenstherapie, die durch Ansätze aus der systemischen Therapie und Achtsamkeitsverfahren ergänzt werden. Insgesamt besteht der Kurs aus zwölf Modulen, welche automatisch basierend auf einem Pre-Assessment aus einem Pool von 17 Modulen ausgewählt werden. Die empfohlene Bearbeitungszeit beträgt zwölf Wochen. Die Module bestehen aus Informationstexten und einer Videomoderation, welche durch das Programm führt und interaktive Aufgaben, Arbeitsblätter und Animationen zeigt. Die Bearbeitungszeit pro Modul wird auf 30 bis 60 Minuten geschätzt. Inhaltlich werden unter anderem Themen wie Psychoedukation, Ressourcen, Abwärtsspiralen, kognitive Umstrukturierung, Entspannung und Achtsamkeit, Aktivitätsaufbau, soziale Beziehungen und

Rückfallprävention behandelt. Die Nutzer haben die Möglichkeit mit einer Nachrichtenfunktion Fragen an Psychologen zu Wochenübungen oder der persönlichen Situation zu stellen. Die Wirksamkeit von Novego wurde bisher in drei randomisiert kontrollierten Studien an unterschiedlichen Patient*innen-Gruppen untersucht. Alle drei Studien wurden in die vorliegende Metaanalyse aufgenommen.

**Novego: Depressionen bewältigen**[1]	
Indikation	Depressive Störung (leicht bis mittelgradig)
Therapieprinzip	KVT, individuell auf die persönliche Lebenssituation abgestimmt
Form der Begleitung	Psycholog*innen-Team kann einmal pro Woche für Fragen und Feedback kontaktiert werden; 24h-Krisenhotline für akute Krisen
Wirkstärke im Vergleich zur Kontrollgruppe	RCT: d = 0,55 (ITT) (180 Teilnehmende) (Beiwinkel et al., 2017); weitere RCTs zur Wirksamkeit bei Schizophrenie (Moritz et al., 2016) und Schmerzpatient*innen mit depressiven Symptomen (Miegel et al., 2019) liegen vor
Möglichkeit der Integration in die f2f-Behandlung	0: DiGA kann unabhängig von der f2f-Behandlung bearbeitet werden
PZN	17865862
Sprachen	Deutsch
Plattformen	Webanwendung

[1] Kontakt: info@novego.de

1.3.6 Selfapys Online-Kurs bei Depressionen

Die von der Selfapy GmbH entwickelte Intervention *Selfapys Online-Kurs bei Depressionen* wurde nach einer Erprobungsphase am 11. April 2022 dauerhaft in das DiGA-Verzeichnis aufgenommen. Der Kurs ist indiziert bei Personen ab 18 Jahren, die an einer mittelgradigen depressiven Störung (ICD-10: F32.1, F33.1) erkrankt sind. Die Intervention basiert auf den Techniken der kognitiven Verhaltenstherapie und beinhaltet die Themenbereiche Psychoedukation, Verhaltensaktivierung, kognitive Umstrukturierung, Problemlösung, Selbstwert und Selbstwirksamkeit, soziale Umwelt, Ressourcen, Achtsamkeit und Rückfallprävention. Die teilweise anhand der Angaben der nutzenden Person individuell angepassten Inhalte werden im Rahmen von zwölf, sequenziell freigeschalteten Lektionen mittels Texten, Audio- und Videoclips sowie interaktiven Übungen vermittelt. Der Hersteller gibt für eine einzelne Lektion eine Bearbeitungszeit von 10 bis 60 Minuten an. Die Nutzung des Kurses kann durch eine/n Psycholog*in überwacht werden, welche/r per Nachrichtenfunktion Fragen zur sachgemäßen Anwendung beantworten kann. Die Wirksamkeit des Online-Kurses bei Depressionen von Selfapy wurde bisher in nur einer randomisiert kontrollierten Studie untersucht, welche in die vorliegende Metaanalyse aufgenommen wurde.

**Selfapys Online-Kurs bei Depressionen**[2]	
Indikation	Depressive Störung (leicht bis mittelgradig)
Therapieprinzip	KVT, u.a. kognitive Umstrukturierung und Verhaltensaktivierung
Form der Begleitung	Psycholog*in steht über Nachrichtenfunktion bei Fragen zur Verfügung (Psycholog*in überwacht zudem zur Wahrung der Patient*innen-Sicherheit im Hintergrund den Kursfortschritt)
Wirkstärke im Vergleich zur Kontrollgruppe	RCT: mit Begleitung: d = 1,63; ohne Begleitung: d = 1,47 (ITT) (401 Teilnehmende) (Krämer et al., 2022)
Möglichkeit der Integration in die f2f-Behandlung	0: DiGA kann unabhängig von der f2f-Behandlung bearbeitet werden
PZN	16954730
Sprachen	Deutsch
Plattformen	Webanwendung

[2]Anforderung von Testzugang: https://www.selfapy.com/fuer-aerzte

Anmerkungen: Abkürzungsverzeichnis und Legende

ACT	Akzeptanz- und Commitment-Therapie
BfArM	Bundesinstitut für Arzneimittel und Medizinprodukte
DiGA	Digitale Gesundheitsanwendung
DGPPN	Deutsche Gesellschaft für Psychiatrie und Psychotherapie, Psychosomatik und Nervenheilkunde
DGP	Deutsche Gesellschaft für Psychiatrie
f2f-Behandlung	Face-to-Face-Behandlung (im persönlichen Kontakt)
GAS	Generalisierte Angststörung
ITT	Intention to Treat
KVT	Kognitive Verhaltenstherapie
NNT	Number Needed to Treat
PMR	Progressive Muskelrelaxation
PP	Per Protocol
PRISMA	Preferred Reporting Items for Systematic Reviews and Meta-Analyses
PZN	Pharmazentralnummer
RCT	randomisiert kontrollierte Studie

Möglichkeit der Integration in die Face-to-Face-Behandlung (f2f-Behandlung)

- 0: DiGA kann unabhängig von der f2f-Behandlung bearbeitet werden
- 1: DiGA kann unabhängig von f2f-Behandlung bearbeitet oder in f2f-Behandlung integriert werden; die DiGA bietet beispielsweise die Möglichkeit einen Report der gesammelten Daten zu erstellen, der an den/die Psychotherapeut*in oder Ärzt*in geschickt werden und gemeinsam besprochen werden kann
- 2: DiGA kann unabhängig von f2f-Behandlung bearbeitet werden, Integration in f2fBehandlung empfehlenswert; es wird beispielsweise empfohlen, dass die Behandler*innen gemeinsam mit den Patient*innen passende Übungen aus dem Übungs-Pool der DiGA heraussuchen oder diese nachbesprechen
- 3: DiGA kann nicht unabhängig von f2f-Behandlung verwendet werden, Integration in f2f-Behandlung notwendig; d.h. Patient*innen können die DiGA nicht ohne eine parallele psychotherapeutische oder ärztliche Begleitung anwenden
